# Amaranth as a Source of Antihypertensive Peptides

**DOI:** 10.3389/fpls.2020.578631

**Published:** 2020-09-25

**Authors:** Agustina E. Nardo, Santiago Suárez, Alejandra V. Quiroga, María Cristina Añón

**Affiliations:** Centro de Investigación y Desarrollo en Criotecnología de Alimentos (CIDCA), Facultad de Ciencias Exactas, Universidad Nacional de La Plata (UNLP), Comisión de Investigaciones Cientíﬁcas (CIC-PBA) and Consejo Nacional de Investigaciones Cientíﬁcas y Técnicas (CONICET- CCT La Plata), La Plata, Argentina

**Keywords:** amaranth, hypertension, bioactivity, peptides, mechanisms of action****

## Abstract

Amaranth is an ancestral crop used by pre-Columbian cultures for 6000 to 8000 years. Its grains have a relevant chemical composition not only from a nutritional point of view but also due to the contribution of components with good techno-functional properties and important potential as bioactive compounds. Numerous studies have shown that amaranth storage proteins possess encrypted sequences that, once released, exhibit different physiological activities. One of the most studied is antihypertensive activity. This review summarizes the progress made over the last years (2008–2020) related to this topic. Studies related to inhibition of different enzymes of the Renin-Angiotensin-Aldosterone system, in particular Angiotensin Converting Enzyme (ACE) and Renin, as well as those referring to potential modulation mechanisms of tissue or local Renin-Angiotensin-Aldosterone system, are analyzed, including *in silico*, *in vitro*, *in vivo*, and *ex vivo* assays. Furthermore, the potential use of these bioactive peptides or products containing them, in the elaboration of functional food matrices is discussed. Finally, the most relevant conclusions and future requirements in research and development of food products are presented.

## Introduction

Amaranth is a pseudocereal that was part of the diet of pre-Columbian cultures. The diet of Aztecs, Mayas, and Incas was mainly based on plant foods, which were low in fat, with moderate protein content and rich in complex carbohydrates. Maya were the first culture to domesticate amaranth about 5000 to 7000 years ago, and to use it as a high-yield crop consumed by all social classes (Bressani, 2003; Montúfar, 2016).

Amaranth grains contain more proteins, lipids, and minerals than many other cereals. The most abundant component, in dry base (d.b) is starch (60–72%), followed by protein (15–19%), lipids (7–8%), dietary fiber (8–16%), and minerals (3–5%). The starch fraction presents a high amylopectin content, the protein fraction has high digestibility (90%) and biological value (86%), the lipid fraction contains unsaturated fatty acids (25:75 saturated:unsaturated) and an unsaponifiable tocopherols and squalene rich fraction, the fiber fraction is rich in xyloglucans and pectic polysaccharides. Amaranth also presents a high percentage of insoluble fiber (78%) and minerals such as copper, manganese, iron, zinc, magnesium, calcium, phosphorus, and potassium. The content of these minerals is higher than that found in rice and corn (Stone and Lorenz, 1984; Bressani, 1994; Alvarez-Jubete et al., 2010; Rastogi and Shukla, 2013; Lamothe et al., 2015; D’Amico and Schoenlechner, 2017; Velarde-Salcedo et al., 2018). The polyphenolic fraction that contains considerable amounts of rutin, isoquercetin, and nicotiflorine is also important (Venskutonis and Kraujalis, 2013; D’Amico and Schoenlechner, 2017). This composition is unique and attractive not only from the nutritional point of view but also as a source of components with good techno-functional properties and enormous potential as bioactive compounds.

Seed proteins are classified as albumins, globulins, glutelins, and prolamines and they determine the structural and physicochemical characteristics of concentrates and isolated. Amaranth proteins have been extensively studied. Globulins, which is the most important fraction, contains 11S and 7S globulins (11S-glob and 7S-glob), as found in other grains (Barba de la Rosa et al., 1996; Martínez et al., 1997; Marcone and Kakuda, 1999; Osuna-Castro et al., 2000; Gorinstein et al., 2001; Quiroga et al., 2010; Quiroga et al., 2012; Tandang-Silvas et al., 2012). Martínez and Añón (1996) have proposed a third fraction, globulin P (P-glob), which is solubilized in water after the extraction of the 11S-glob and 7S-glob in saline solution. The P-glob fraction had previously been identified by Konishi et al. (1991) who had named it albumin-2. P-glob is composed of unitary molecules of molecular weight and polypeptide composition similar to those of 11S-glob and aggregates larger than 500 kDa. It also contains a 56 kDa distinctive polypeptide (an unprocessed subunit), which is involved in the polymer stabilization (Martínez and Añón, 1996; Martínez et al., 1997; Molina et al., 2008). These features make P-glob a hallmark of amaranth. Besides, P-glob has a less compact and more hydrophobic structure than that of 11S-glob; it is thermostable and presents a high degree of polymerization (Castellani et al., 1999; Castellani et al., 2000).

According to Quiroga et al. (2007, 2009); 11S-glob and P-glob would be two 11S-glob isoforms formed by polypeptides encoded by two different legumin gene subfamilies. Velarde-Salcedo et al. (2018) have recently suggested that amaranth glutelins could be polymerized globulins.

Different studies have shown that amaranth proteins, as protein isolates or isolated fractions, have good gelling, foaming and emulsifying capacities and presents good solubility at acidic and alkaline pH (Cordero-de-los-Santos et al., 2005; Avanza et al., 2006; Bejarano-Luján et al., 2010; Ventureira et al., 2012; Bolontrade et al., 2013; Tapia-Blácido et al., 2013; Condés et al., 2013; Shevkani et al., 2014a; Shevkani et al., 2014b; Bolontrade et al., 2016; López et al., 2018; Suarez and Añón, 2018). On the other hand, it has recently been demonstrated that amaranth storage proteins, like other proteins of plant and animal origin, have encrypted amino acid sequences displaying different physiological activities: antihypertensive, antioxidant, antiproliferative/antitumor, antithrombotic, antihaemolitic, antimicrobial, hypocholesterolemic, hypoglycemic, and immunomodulatory. These sequences can be released by enzymatic treatment, through fermentative processes or during the gastrointestinal digestion (Barrio and Añón, 2010; Lado et al., 2015; Sabbione et al., 2016a; Sabbione et al., 2016b; Moronta et al., 2016a; Moronta et al., 2016b; Orsini Delgado et al., 2016; García Fillería and Tironi, 2017; Quiroga et al., 2017; Orona-Tamayo et al., 2019; Martinez-Lopez et al., 2020; Nardo et al., 2020; Suárez et al., 2020).

Bifunctionality (technological and biological) and nutritional properties make amaranth a promising and very interesting source for the formulation of functional foods.

## Bioactive Peptides as Blood Pressure Regulators

Cardiovascular diseases (CVD) are one of the most important chronic non-communicable diseases and one of the leading causes of death in the world, even doubling those corresponding to cancer (WHO, 2016). The main risk factor for CVD is hypertension, which is responsible for 45% of heart attacks and 51% of strokes (WHO, 2018).

Pharmaceutical industry has developed different medications to control hypertension; some of them act on components of the Renin-Angiotensin-Aldosterone system. Among these drugs are angiotensin converting enzyme (ACE) and renin inhibitors, angiotensin II Type-1 receptor (AT1 receptor) blockers, calcium channel blockers, alpha and beta-blockers, diuretics, and vasodilators. An interesting alternative therapy is to control blood pressure (BP) by means of diets, including the consumption of functional foods, thus reducing the risk of developing non-communicable diseases, such as cardiovascular diseases. Although there are different components present in foods with proven functional or physiological activity, bioactive peptides have become relevant in recent years.

Particularly, antihypertensive peptides have been found and characterized in animal and vegetable proteins from different food sources (milk, fish, meat, eggs, and different vegetables, among others). Most of them are ACE inhibitors although, renin inhibitors and NO production activators have been described (Hernández-Ledesma et al., 2011; Martínez-Maqueda et al., 2012; Hernández-Ledesma et al., 2013; He et al., 2014; Aluko, 2015a; Saleh et al., 2016; Borghi and Cicero, 2017; Cicero and Colletti, 2017). Among different food sources of antihypertensive peptides, dairy proteins are the most studied including trials in humans (Cadée et al., 2007; Martínez-Maqueda et al., 2012). Besides, commercial antihypertensive products have been developed as Peptide C12 which contains a casein-derived peptide with sequence FFVAPFPGVFGK, this peptide reduces BP in pre-hypertensive people (Cadée et al., 2007).

Due to the worldwide relevance of CDV and the possibility of using bioactive peptides as an alternative and complementary therapy, it is important to deepen our understanding of the blood pressure control system in humans.

### RAAS System

**Figure 1:**
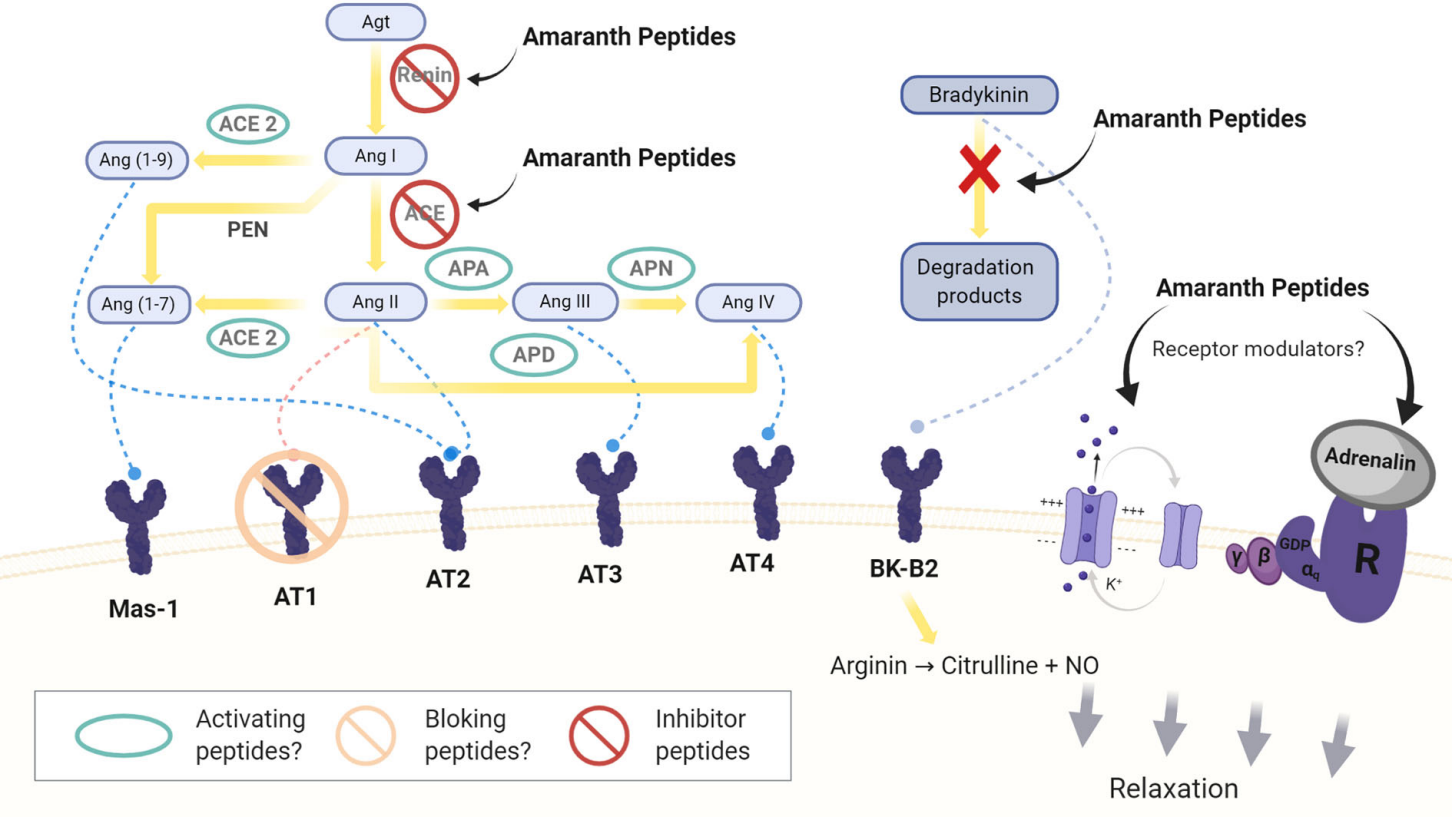
Sites of action of amaranth peptides in the circulating and local RAAS system. Agt: angiotensinogen; Ang I, II, III, IV, (1-9), (1-7): angiotensin I, II, III, IV, (1-9) and (1-7); ACE: angiotensin converting enzyme; APA, APN, and APD: aminopeptidases A, N, and P; PEN: neutral peptidases. Mas-1: Mas-1 receptor, AT1: angiotensin II type1 receptor, AT2: angiotensin II type 2 receptor, AT3: angiotensin III receptor AT4: angiotensin IV receptor. BK-B2: bradykinin B2 receptor.

The Renin-Angiotensin-Aldosterone system, RAAS, is a complex dual system acting at systemic and local levels (**Figure 1**). The ***Classic circulating RAAS*** plays a role in the regulation of cardiovascular function through its contribution to the maintenance of BP and fluid balance. The ***Tissue/local RAAS*** is present in several organs, the lymphatic system, the adipose tissue, and blood vessels. These tissues can synthetize the components of the circulating RAAS and form **Ang II** independently from the circulating system (Carey and Siragy, 2003; Nguyen Dinh Cat and Touyz, 2011; Zhuo et al., 2013).

In the RAAS, Ang II is formed from angiotensinogen (**Agt**) by the action of two enzymes, **renin** (Inagami, 1981) and **ACE** (Corvol et al., 2004; Zhuo et al., 2013). Agt is transformed into angiotensin I (**Ang I**), which is rapidly converted to Ang II. Ang II is a potent vasoconstrictor that acts on vascular cells of the smooth muscle through its binding to the **AT1 receptor** but it also interacts with the angiotensin II type 2 receptor (**AT2 receptor**) decreasing BP (Unger et al., 1996; de Gasparo et al., 2000). As well Ang II increases sodium reabsorption and stimulates the aldosterone and vasopressin production (Touyz and Schiffrin, 2000).

Ang II, *via* AT1 receptors, acts on the endothelium, whose activity is regulated by the cellular redox state (Oskarsson and Heistad, 1997; Jiang et al., 2008). High levels of Ang II lead to oxidative stress and a decrease of NO through the activation of the NADPH/NADH oxidase. This leads to inflammation and cell growth. Ang II also stimulates the release of endothelin-1 (ENT-1), which is a potent vasoconstrictor (Pérez et al., 2003).

More recent studies assessing the local RAAS have identified new components and mechanisms, some of which contribute to the natriuretic/vasodilator effects of the system by opposing the effects of Ang II (Carey and Siragy, 2003; Zhuo et al., 2013). Different peptidases transform Ang II in less potent or inactive peptides, **Ang III** and in a **Ang IV** (Padia et al., 2006). Ang IV binds to the **AT4 receptor**, which is expressed in different tissues and exerts different actions as increasing the renal cortical blood flow, the tubular reabsorption of Na^+^ and natriuresis (Handa et al., 1998; Albiston et al., 2001).

Ang II is also hydrolyzed by ACE2, which hydrolyzes Ang I, but to a lesser extent (Donoghue et al., 2000; Turner et al., 2002; Hamming et al., 2007). The product of this hydrolysis is **Ang 1-7**, an heptapeptide with vasodilator capacity that binds to the **Mas-1 receptor** (Ferrario et al., 1991; Santos et al., 2003; Kostenis et al., 2005; Santos et al., 2013). The hydrolysis of Ang I can in turn generate **Ang 1-7** by the action of neutral tissue endopeptidases, **NEP 24.11, 24.15 and 24.26,** or it can be converted into **Ang 1-9**, which is then converted into Ang 1-7 by ACE (Yamamoto et al., 1992; Vickers et al., 2002; Ferrario, 2006; Zhuo et al., 2013).

A specific receptor for renin and prorenin (**PRR**) has been detected in different tissues. PRR would exert Ang II-dependent and independent effects (Nguyen, 2007; Advani et al., 2009).

New insights into the RAAS system have provided novel targets to control BP, particularly through the inhibition of key enzymes, receptor or other mechanisms.

### Antihypertensive Peptides in Amaranth Seeds

The first studies related to the existence of bioactive peptides encrypted in amaranth storage proteins date to a decade ago. Since then, different types of *in vitro, in vivo, ex vivo* and *in silico* tests have been carried out, which have allowed obtaining additional information and building a still incomplete painting.

#### ACE - Inhibitory Peptides

ACE is one of the main targets in the search for peptides with antihypertensive activity, either as a replacement or as a complement to the standard drugs. **ACE (**EC 3.4.15.1) is a membrane-bound dicarboxypeptidase that requires Zn^++^ and Cl^-^ as cofactors. This enzyme exists in two isoforms: the somatic enzyme (**ACEs**) and the testicular enzyme (**ACEt**). Two forms of the somatic enzyme can be identified, the tissue and the soluble forms. **ACEs** is the main producer of Ang II and is expressed in most human tissues including vascular endothelial cells. It is a highly glycosylated protein with two metalloproteinase domains, N- and C-, each containing a catalytic site with the canonical Zn^2+^ binding motif HEXXH (Watermeyer et al., 2009; Bernstein et al., 2013).

**ACE** catalyzes the conversion of the Ang I decapeptide (DRVYIHPFHL) into a potent vasoconstrictor, Ang II, by the removal of the two C-terminal amino acids. Although Ang I has the same affinity for both catalytic sites, the generation of Ang II is mainly accomplished by the C-domain. The catalytic efficiency of the C-domain is three times higher than that of N- domain (Van Esch et al., 2005; Fuchs et al., 2008; Masuyer et al., 2012).

**ACE** is also part of the kinin-kallikrein system that degrades the potent vasodilator bradykinin to yield inactive fragments. The latter reaction is catalyzed equally efficiently by both the C- and the N-domain (Corvol et al., 2004). These evidences clearly indicate that each domain presents substrate specificity. Therefore, to accomplish the inhibition of ACE, peptides capable of specifically interacting with the C- domain are required.

These results clearly indicate that the hydrolytic activity of each domain has substrate specificity. Therefore, if one wishes to inhibit ACE, one should look for peptides capable of specifically interacting with the C-domain of the enzyme and/causing binding of conformational changes at the active site leading to a lack or decrease of recognition of the natural substrate.

In the particular case of amaranth, numerous studies have been carried out which will be discussed depending on the type of analysis performed.

#### *In Silico* Assays

*In silico* studies carried out by Silva-Sánchez et al. (2008) have demonstrated for the first time the existence of different bioactive peptides in amaranth storage proteins. The search for bioactive peptides was done by exploring two databases, one corresponding to the amaranth protein sequence (www.ncbi.nlm.nih.gov) and the other comprising existing bioactive peptides (actually BIOPEP-UWM, http://www.uwm.edu.pl/biochemia/index.php/pl/biopep). Of the twelve potential activities detected (activating ubiquitin mediated proteolysis, antiamnestic, antihypertensive, antioxidant, antithrombotic, embryotoxic, immunomodulating, immunostimulating, ligand, opioid, regulating, protease inhibiting), antihypertensive one was the most frequent in 11S-glob protein. Using the same type of analysis, it was located in the sequence of this protein di and tripeptides, five of which (GKP, LF, YL, RF, and HY) according to the literature were ACE inhibitors (**Table 1**).

**Table 1:** Sequences of bioactive peptides derived from Amaranth proteins with antihypertensive activity.

Sequence	Level of evidenceof bioactivity	Mechanismof action	IC_50_/*EC_50_*	Reference
GKP	Database search	ACE inhibitor	*0.352 mM*	Silva-Sánchez et al., 2008
LF			*0.349 mM*	
FP			*0.315 mM*	
YL			*0.122 mM*	
RF			*0.093 mM*	
HY			*0.026 mM*	
VY			*0.007 mM*	
VW			*0.001 mM*	
LPP, LRP, VPP, YP	Identified in an active fraction	ACE inhibitor		Silva-Sánchez et al., 2008
ALEP	Evaluated *in silico* and *in vitro*. In bold peptide evaluated *in vivo.*	ACE inhibitor	6.32 mM	Vecchi and Añón, 2009
VIKP			0.175 mM	
AY, FP, FY, GY, HY, IR, IY, LF, LW, LY, MF, MY, PR, RF, RL, RY, VF, VW, VY, YG, YL,YP, AAP, AIP, AVP, FNQ, FQP, GGY, GKP, GRP, HIR, IKP, ILP, IRA, LAA, LAY, LLP, LNP, LPP, LQP, LQQ, LRP, LSP, LVL, LVR, PLP, PQR, PRY, TAP, VAA, VAP, VAY, VLP,VPP,VRP, VSP, VYP, ALPP, LAMA, YGGY	Identified in glutelin hydrolysates. In bold are show peptides previously reported in databases as ACE inhibitors.	Induce endothelial NO production and or/ACE inhibitors		Barba de la Rosa et al., 2010
YESGSQ, GGEDE, QQQLV, ACDIP, FLISCLL, TALEPT, HVIKPPS, SVFDEELS, ASANEPDEN, VEEEGNM, DFIILE, EVEAAI	Identified in an active fraction.	ACE inhibitor		Vilcacundo et al., 2019
NIDMLRL, LVRW, VRWS, VR, CIHNIVY	Identified in an active fraction.	ACE inhibitor		Ayala-Niño et al., 2019a
FNLPILR	Identified in an active fraction. Evaluated *in silico* and *in vitro.*	Renin inhibitor	0.41 mM	Quiroga et al., 2017 Nardo et al., 2020
SFNLPILR			2.50 mM	
AFEDGFEWVSKF			1.47 mM	

Using bioinformatics tools, Vecchi and Añón (2009) identified two tetrapeptides in the sequence of the 11S-glob, namely VIKP and ALEP. These tetrapeptides displayed inhibitory capacity on ACE. The analysis consisted of different stages. Initially, the authors performed a molecular modeling of 11S globulin and then they examined the potential water accessibility (accessible surface area, ASA) of each amino acid residue and identified the encrypted antihypertensive peptides according to the information of BIOPEP-UWM database. Considering that the functionality of bioactive peptides depends on their release from the mother sequence, Vecchi and Añón (2009) identified two tripeptides, IKP and LEP with high antihypertensive capacity. These peptides were located on the surface of the protein molecule. From KP and EP, two libraries were constructed by N-terminal extension using the 11S-glob primary sequence, employing ligand-protein docking assay to simulate the ability of each member of the library to bind the active site of ACE. Most of evaluated peptides were found to be suitable ligands, displaying interaction energy values that were comparable to that of control inhibitory peptides (KP, IKP, EP, LEP and captopril). In all cases, the peptides interacted through ionic bonds established with the free coordination sites of the Zn^++^ of the active site. VIKP and ALEP were the peptides with the highest potential antihypertensive activity, being their theoretical interaction free energy of a magnitude comparable to that of the parental peptide. Both peptides can also be found in *Glycine max* glycinin and in the *Chenopodium quinoa* 11S-glob.

**Figure 2:**
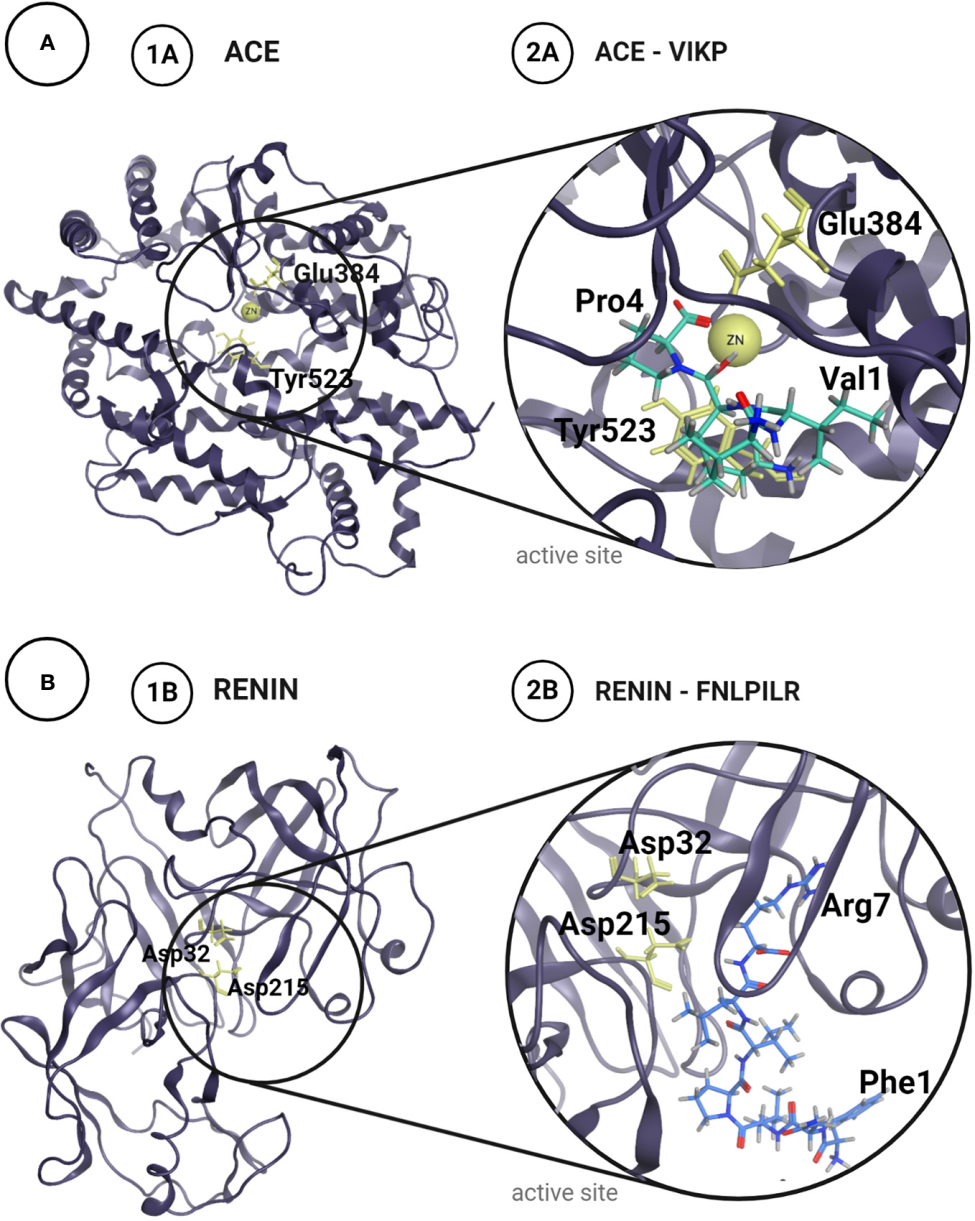
Structures of Angiotensin Converting Enzyme, ACE **(A)** and Renin **(B)** with their catalytic residues marked in yellow (1A, B). Proposed complex for the most potent inhibitor peptides identified in amaranth proteins for each enzyme (2A, B).

Vecchi and Añón (2009) confirmed the predicted results obtained *in silico* by synthesizing the two tetrapeptides and verifying their ACE inhibitory activity *in vitro.* Both ALEP and VIKP presented inhibitory capacity, with IC_50_ values of 6.32 mM and 175 µM, respectively (**Table 1**). The difference in the IC_50_ values could be attributed, at least partially, to the way in which these peptides bind to the active site: VIKP interacts with the Zn^++^ ion *via* its C-terminal carboxyl moiety, while ALEP does so by means of its glutamic acid R group, as shown in **Figure 2A**. This work was the first one to use a peptide docking approach to search for peptides with antihypertensive activity and to assess their ACE inhibition capacity.

Quiroga et al. (2012) analyzed the potential bioactivity of the 7S globulin. These authors performed a theoretical search by comparing the tryptic sequences of the 7S-glob subunits of 66, 52, 38, 35, and 16 kDa previously identified with the ACE inhibitor sequences in the BIOPEP-UWM database. In all subunits they found numerous di and tripeptides with potential antihypertensive activity.

The use of bioinformatics tools has allowed demonstrating that amaranth storage proteins and their constituent fractions are potential sources of antihypertensive peptides and to identify two tetrapeptides encrypted in the 11S-glob as competitive ACE inhibitors.

#### *In Vitro* Assays

Tovar-Pérez et al. (2009) studied the ACE-inhibitory capacity of the peptidic fraction obtained by alcalase hydrolysis of albumins and globulins of *A. hypochondriacus*. The hydrolysis of albumin and globulin, carried out for 15 and 18 h, respectively, yielded different maximum degrees of hydrolysis, DH, 44 and 13%, respectively. These hydrolysates showed a moderate ACE inhibitory capacity. The greatest inhibition corresponded to the globulin fraction (IC_50_ 1.16 and 0.63 mg/ml, for albumins and globulins, respectively), which agrees with the predictive analysis performed by Rastogi and Shukla (2013). The hydrolysates were subsequently purified by filtration gel chromatography and RP-HPLC, obtaining fractions with an average size of 3–4 amino acids and IC_50_ values of 0.35 and 0.15 mg/ml for albumins and globulins, respectively.

By LC/MM/MM analysis of tryptic digests of the glutelin fraction of *A. hypochondriacus*, Silva-Sánchez et al. (2008) also detected the sequences LPP, LRP, VPP, and YP. Aoyagi (2006) demonstrated that the same sequences from buckwheat, another pseudocereal, presented ACE inhibitory activity in spontaneously hypertensive rats.

Studies carried out with concentrates of *A. cruentus* hydrolyzed with alcalase have shown that the peptides obtained had ACE inhibitory activity (IC_50_ 0.176 ± 0.014 mg/ml) (Tiengo et al., 2009). This capacity remained almost unaltered when the concentrates were thermally treated before hydrolysis (EC_50_ 0.138 ± 0.002 mg/ml), suggesting that protein denaturation does not affect the sensitivity to alcalase hydrolysis.

Fritz et al. (2011) used different proteases—pronase, papain, trypsin, chymotrypsin and alcalase—and different assay conditions to obtain hydrolysates from the protein isolate of *A. mantegazzianus*. In most cases, the hydrolysates contained ACE inhibitor peptides. However, the hydrolysate with the highest activity was that obtained with alcalase (0.8 mg/ml - 4 h - 37 °C). The degree of inhibitory activity obtained was proportional to the degree of hydrolysis, suggesting a relationship between this activity and peptide length. The IC_50_ values obtained in *in vitro* assays for hydrolysates with 45 and 65% DH were 415 and 600 μM, respectively, the same order of magnitude than those usually found for food protein hydrolysates. Fritz et al. (2011) stated that these IC_50_ values were similar to those obtained with the pure antihypertensive inhibitors included in the BIOPEP-UWM database at the time, suggesting the presence of peptides with different inhibitory potency in the hydrolysate.

Ayala-Niño et al. (2019a) analyzed the profile of peptides obtained by the consecutive hydrolysis of a protein isolate from *A. hypochondriacus* with alcalase and flavourzyme. Their results showed that action of both enzymes affords hydrolysates with high antioxidant, antithrombotic and antihypertensive activity. It should be noted that the values obtained by Ayala-Niño et al. (2019a) are not very different from those reported by other authors for hydrolysates with alcalase (Fritz et al., 2011; Ramírez-Torres et al., 2017). The chromatographic fractionation of the hydrolysates obtained with alcalase-flavourzyme rendered 14 fractions out of a total of 56, in which known proteins were identified. The IC_50_ ranges for these fractions were similar to those of hydrolysates obtained under other experimental conditions. Finally, the sequence identification of the peptides presents in two of these fractions was performed by MALDI-TOF. This analysis allowed identifying novel peptide sequences 4–8 amino acids long, such as NIDMLRL, LVRW, VRWS, VR, and CIHNIVY, containing amino acids and short sequences whose ACE inhibitory capacity had been demonstrated in other systems (Ueno et al., 2005; De Gobba et al., 2014; Aluko, 2015b; Balgir and Sharma, 2017) (**Table 1**).

Taking into account these results and those obtained in other food systems, it can be suggested that alcalase is the enzyme of choice to release bioactive peptides from amaranth storage proteins. Recently Ramírez-Torres et al. (2017) optimized the process conditions using a commercial protein isolate as substrate. To do this, authors used a response surface analysis with 24 possible combinations of reaction conditions (pH, reaction time, enzyme concentration, and temperature) and in all cases ACE inhibitory capacity was evaluated. The results obtained in those experiments indicated that the optimal conditions for hydrolysis with alcalase were pH 7.01, 52°C, enzyme concentration 0.04 mU/mg, and 6.16 h. The degree of hydrolysis reached was 74.77% with a 93.5% of ACE inhibition.

Fermentation was also evaluated as a method to release of bioactive peptides encrypted in amaranth storage proteins. Ayala-Niño et al. (2019b) used *Lactobacillus casei* Shirota and *Streptococcus thermophilus* 54102 in mono- and combined culture as starters. The authors detected the release of antihypertensive peptides (45% inhibition of ACE).

Unlike those discussed so far, Luna-Suárez et al. (2010) used an alternative route to obtain an amaranth protein fraction, 11S-glob, with increased antihypertensive activity. These authors used genetic engineering tools to insert four VP sequences, of recognized antihypertensive action, in tandem in the fourth variable region of the molecule. The hydrolysates obtained by the action of trypsin-chymotrypsin showed a higher activity, eight times higher, (IC_50_ 0.06 mg/ml) than that corresponding to the hydrolysate obtained from unmodified 11S-glob. The expression of this protein in *E.coli* showed a high degree of insolubilization and the highest amount of recombinant protein expressed was 60 mg/L. In order to improve the functionality of this protein, a second modification was introduced in the C-terminal zone (Castro-Martínez et al., 2012). In this case, the maximum production of the recombinant protein obtained was 99 mg/L and the IC_50_ of ACE inhibition of its hydrolysate was 0.047 mg/ml.

Recently, Espinosa-Hernández et al. (2019) introduced a modification in 11S-glob, adding four VY with antihypertensive properties in the C-terminal region of the molecule. This new protein has a higher level of expression in *E.coli*, presents better structural stability and its trypsin hydrolysate, a better ACE inhibitor potential (IC_50_ 0.034 mg/ml). Although IC_50_ values for ACE inhibition are higher than those indicated for peptides obtained by hydrolysis of 11S globulin, glutelins, isolates or concentrates, an in-depth analysis is required regarding costs, preparation times, and possibilities of use in food.

The studies presented above show that the different protein fractions that make up the storage proteins of the amaranth grain, as well as derived protein products, isolates and protein concentrates, have encrypted peptides with ACE inhibitory activity *in vitro*. These results also indicate that proteases from microorganisms can be used to release bioactive peptides.

These evidences, however, imply that neither the release of these active peptides invariably occurs *in vivo*, nor that, in case such release is feasible, the active peptides will be bioavailable to exert their biological function.

Different studies have used a ***simulated gastrointestinal digestion*** process to analyze the accessibility of ACE-inhibiting amaranth peptides. Initially, simulation protocols involved the sequential use of pepsin and trypsin, but the experimental conditions varied among authors, which made the comparison of results extremely difficult. Most of the research groups have recently adopted the consensus protocol developed within the framework of the INFOGEST Program (Minekus et al., 2014), which will allow a better interpretation of the results obtained.

Tiengo et al. (2009) employed a hydrolysis protocol with pepsin-trypsin to simulate the gastric digestion process of unheated and heated concentrates of *A. cruentus* and its alcalase hydrolysates. The highest values of IC_50_ were those obtained with unheated and heated concentrates (0.439 ± 0.018 mg protein/ml and 0.475 ± 0.021 mg protein/ml, respectively) and the lowest value corresponded to alcalase hydrolysates, before and after a simulated DGI (0.137 ± 0.002 mg protein/ml, and 0.176 ± 0.014 mg protein/ml, respectively). Under those experimental conditions, the results suggest that the degree of protein denaturation does not affect the sensitivity to alcalase hydrolysis, that the peptides released by alcalase are resistant to the action of the simulated gastrointestinal digestion process and that the antihypertensive peptides can be released during the DGI of amaranth concentrates, although with reduced activity.

Only one study has demonstrated the ACE inhibitory capacity of a partially purified 7S-glob subjected to a simulated DGI, with this activity being similar to that of the 11S-glob digests (EC_50_ 0.17 ± 0.03 mg/ml and 0.17 ± 0.01mg/ml, respectively) (Quiroga et al., 2012).

Aphalo et al. (2015) reported the ACE inhibitory activity of amaranth sprouts and demonstrated that this activity increases after a simulated DGI (IC_50_ 0.26 ± 0.07 mg/ml).

Vilcacundo et al. (2019) have recently subjected a protein concentrate of *A. caudatus* to a gastrointestinal digestion process using the protocol published by Minekus et al. (2014) and analyzed the potential multi-functionality of the released peptides in each digestion stage. These authors evaluated the antioxidant and the ACE inhibitory capacities, the viability of colon cancer cells and the ability to inhibit DPP-IV, α-amylase. The IC_50_ values for ACE inhibitory capacity were 79.13 ± 1.08, 39.00 ± 2.99, and 88.01 ± 13.96 µg/ml for the initial concentrate, after gastric digestion and at the end of the process, respectively. These results indicate that during the gastric stage, peptides with greater inhibitory capacity are released; and that these peptides would be sensitive to pancreatic enzymes. Vilcacundo et al. (2019), compared their results with those obtained by Soriano-Santos and Escalona-Buendía (2015) with alcalase hydrolysates of amaranth albumins and globulins and concluded that the alcalase hydrolysis promoted the release of inhibitory peptides, but to a lesser extent than the simulated digestion. Comparing the results obtained by Vilcacundo et al. (2019) and Fritz et al. (2011), is possible to infer that differences the ACE inhibitory activity obtained in both studies are more similar, suggesting that the inhibition values obtained depend not only on the type of enzyme used but also on the experimental conditions. By LC/MS/MS these authors identified the peptides released at the end of the simulated DGI. These peptides, 13 sequences, consisted of 5–9 amino acids long sequences, which matched amaranth proteins found in the database. Among the peptides released, eight contained leucine, proline, valine, histidine, or phenylalanine, which could be crucial to exert the ACE inhibitory effect (**Table 1**).

These studies clearly demonstrate not only the existence of amaranth peptides with the ability to inhibit ACE, but also that many of these peptides are resistant to the hydrolytic action of digestive enzymes.

In most of the *in vitro* studies performed the specific peptide/s responsible for the activity is unknown and when putative sequences were identified their activity was not experimentally verified. Therefore, despite the relevant advances made in the last ten years by different research groups, many aspects remain to be clarified.

#### *In Vivo* Assays

The biological activity of peptides is dependent not only on their accessibility, but also on their bioavailability. In this sense, transport experiments must be carried out both *in vitro* and *in vivo*.

Fritz et al. (2011) demonstrated for the first time the antihypertensive capacity of protein hydrolysates of *A. hypocondriacus* *in vivo* using spontaneously hypertensive Wistar rats (SHR). The intragastric administration of alcalase hydrolysates with 45% DH was effective in lowering blood pressure values of SHR rats in a dose-dependent fashion. The hypotensive effect was maximal 90 min after administration of the hydrolysate and persisted for up to 7 h. In these experiments, BP dropped from 140 to 100 mmHg. These results support an action of the amaranth peptides on the vascular peripheral resistance through the inhibition of ACE, as demonstrated *in vitro*. In order to rule out a possible effect of amaranth peptides on the myocardium, which would affect the cardiac output, Fritz et al. (2011) carried out experiments on papillary muscles isolated from SHR. In these experiments, authors were not able to detect any significant negative inotropic effect. These authors also performed *ex vivo* experiments performed on isolated aortic rings have shown that the contraction induced by norepinephrine (10^-8^ M – 10^-4^ M) is antagonized by amaranth peptides (0.1 g/ml), suggesting a vasodilator effect. These results would support the idea that the amaranth peptides present in the hydrolysate would induce the reduction of BP by decreasing the vascular peripheral resistance, even in isolated systems such as the aorta, suggesting that the hydrolysate would exert its effect on both the local and tissue RAAS.

Lado et al. (2015) corroborated the antihypertensive effect of amaranth proteins/peptides *in vivo*. These authors reported that normotensive Wistar rats fed for 4 weeks with a normal diet for laboratory rodents (AIN93), in which 12.5% of casein had been replaced by amaranth protein isolate, experienced a reduction in their BP of 27 mmHg, as compared to the control group fed only with casein as a protein source.

Ramírez-Torres et al. (2017) analyzed the bioavailability of alcalase amaranth protein hydrolysates in BALB/c mice. Three groups of animals received intragastric water, captopril (25 mg/kg animal) and the amaranth hydrolysate (2.4 g/kg animal), respectively. Blood samples were then collected from the tail vein (5–60 min) and indirectly determined the presence in the serum of amaranth peptides with ACE inhibitory activity. After 5 and 10 min, the groups administered with captopril and the hydrolysate showed a high percentage of ACE inhibition (approximately 50 and 30% inhibition, respectively), and this inhibition was maintained for approximately 60 min. Throughout the experiment, the group that received water showed an ACE inhibition equal to or less than 10%. After 120 min, all the animals presented very low and similar inhibition values. SHR rats, which received the same treatment as BALB/c mice, were used to determine systolic BP values. The animals’ systolic BP (approx. 180 mmHg) began to decrease at 3 h after the administration of either captopril of the hydrolysate, reaching the lowest value after 5 h (110 and 140 mmHg for animals treated with captopril or the hydrolysate, respectively).

**Figure 3:**
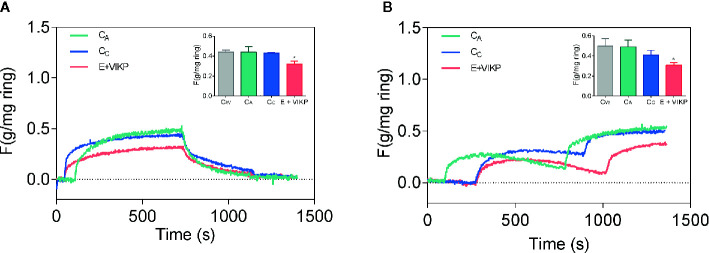
Effect of different samples on contractile activity of isolated aortic rings as the strength per weight of the ring (gF/mg) contracted by exposure to potassium **(A)** and norepinephrine **(B)**. Rings extracted from animals treated with emulsion added with VIKP (E+VIKP), captopril (CC) aliskiren (CA) and water (CW).

Suárez et al. (2020) have recently demonstrated that different products derived from amaranth, a protein isolate, an alcalase hydrolysate, VIKP (a peptide with proven antihypertensive activity, or an oil-water emulsion formulated with isolate-hydrolyzed amaranth as a surfactant with and without the addition of VIKP), have antihypertensive activity in SHR. It should be noted that the amount of sample supplied to the animals was 38 times lower in the case of VIKP (50 mg/Kg rat vs 1.9 g protein/Kg rat). The results obtained in these experiments indicate that the administration of amaranth-based emulsions, either with and without the addition of VIKP, cause a reduction in the systolic BP of about 42 and 35 mmHg. This reduction of BP was equal to or greater than that induced by captopril (35 mmHg), a recognized ACE inhibitor. The rest of the samples tested induced a drop in systolic BP of 26–21 mmHg, showing that VIKP is capable of reducing BP to a much lower concentration than API and APH. All the samples tested, like captopril, induced an increase in the amount of circulating ACE, whose activity was inversely proportional to its concentration. This suggests a greater release of ACE to the circulatory system but with minimal activity. These authors also determined the contractile activity of thoracic aorta rings in the presence of potassium ions and norepinephrine. These compounds are known to cause vasoconstriction through the action on voltage- and receptor-operated channels, respectively. In the presence of potassium, the contractile activity of the aorta rings from the animals treated with all the samples was lower, while in the presence of norepinephrine, less activity was detected only in the aortas from animals administered with VIKP dissolved in water or added to the emulsion made from amaranth. It is noteworthy that no reduction in the contractile activity was detected in animals treated with captopril, a competitive ACE inhibitor. **Figure 3** shows the contractile intensity of isolated aortic rings contracted by exposure to potassium (**Figure 3A**) and norepinephrine (**Figure 3B**). According to these results, the amaranth peptides released during the gastrointestinal digestion process *in vivo* and/or resistant to such process would not only inhibit ACE but would also act on the local RAAS system, affecting the polarity of the membrane and, possibly in the case of VIKP, interacting with adrenergic receptors.

The results discussed so far strongly suggest that the amaranth peptides and/or products thereof generated in the gastrointestinal digestion process are bioavailable and exert a marked reduction in blood pressure in hypertensive animals.

### NOs Peptides

As mentioned, ACE is an enzyme capable of interacting with a variety of substrates, such as Ang I and bradykinin, among others. Bradykinin (RPPGFSPFR) has a potent vasodilator and inflammatory action. It is capable of binding to two receptors, BK-B1 and BK-B2, belonging to the G protein coupled receptor (GPCR) family. Its binding to the B2 receptor induces an increase in intracellular Ca^++^ in the endothelium and activates one of the isoforms of nitric oxide oxidase (eNOs). In the presence of oxygen, this enzyme transforms arginine into citrulline and NO, which acts on the smooth muscle stimulating the guanylate cyclase, the enzyme responsible for transformation of GTP into GMPC, which is a mediator with vasodilator properties. GMPC is in turn transformed into GMP and pyrophosphate by the action of GMP phosphodiesterase. NO is also activated by acetylcholine and growth factors like the vascular endothelial growth factor (Förstermann and Li, 2011).

Barba de la Rosa et al. (2010) analyzed the capacity of peptides encrypted in the sequence of glutelins released by the action of trypsin to inhibit ACE and the production of NO. These authors identified more than 60 peptides in the hydrolysate, mostly tri and dipeptides, some of which with recognized antihypertensive activity (YP, LPP, LRP and VPP). The hydrolysate presented an IC_50_ value of 200 µg/ml for ACE inhibition (**Table 1**). Authors hypothesized that the inhibition of ACE would lead to an increase in bradykinin levels and therefore the hydrolysate obtained could induce an increase in NO production. Their results showed that both the hydrolysate obtained and the intact glutelins induced NO production, with the increase induced by the hydrolysate being of a greater magnitude (97 µM and 75 µM for 100 µg/ml of sample, respectively). In order to determine if the hydrolysate was able to stimulate NO production through activation of eNOS, rat coronary endothelium cells were incubated with either bradykinin, a bradykinin receptor inhibitor (HOE-140) and the hydrolysate. They observed that both bradykinin and the hydrolysate stimulated NO production and that this production was totally inhibited by HOE-140 in the case of bradykinin and only partially inhibited in the case of the hydrolysate. Thus, the eNOs activity induced by hydrolysate is mediated, in part, by the BK-B2 receptor; and the participation of other signaling pathways in control of vascular activity cannot be ruled out. To corroborate the NO production, the authors examined the capacity of hydrolysate to induce vasorelaxation of aortic rings. The hydrolysate induced vasorelaxation of rings in a dose-dependent manner, an effect that was partially blocked when the endothelium was removed from the vessel. The hydrolysate-induced vasorelaxant effect was totally inhibited when the rings were pretreated with HOE-140, suggesting a central role for bradykinin.

Fritz et al. (2011) also performed *ex vivo* experiments using aortic smooth muscle rings as discussed above. The exposure of aortic rings to the amaranth hydrolysate previously contracted with norepinephrine causes a significant decrease in the contractile response. These authors reported that the preincubation of aortic rings with 100 mM N(ω)-nitro-L-arginine methyl ester, L-NAME, a widely used eNOs inhibitor, did not cause any change in the response. This suggests that the vasorelaxant effect observed with the peptides derived from the globulin fraction present in the hydrolysate would not be associated with the production of NO.

### Renin Inhibitory Peptides

Renin (EC 3.4.23.15) is a 37 kDa endo aspartyl protease that belongs to peptidase A1 family that is secreted by the kidney into the bloodstream (Inagami, 1981; Yuan et al., 2006; Pihlanto and Mäkinen, 2017; Aluko, 2019). The most important structural feature of renin is the antiparallel beta sheet that forms two similar lobes/domains with a cleft between them. The active site is located in the cleft and substrate recognition site consists of 5 pockets, namely S1, S2, S3, S1′ and S2′, and a subpocket termed S3^SP^ (Rahuel et al., 2000). Its active site contains two Asp that are used to cleanse the substrate through an acid-base hydrolysis mechanism.

Quiroga et al. (2017) studied for first time the capacity of amaranth peptides to inhibit renin, the first enzyme of the RAAS system. The authors obtained alcalase hydrolysates from protein isolates of *A. hypocondriacus* with an average peptide length of 4–6 amino acids (HD 21%) and a molecular mass range of 1.355 and 0.179 kDa corresponding to 12 amino acid long peptides and free amino acids. These peptides were able to inhibit renin, reaching 60% inhibitory capacity and an IC_50_ of 0.6 mg/ml. These values were similar to those reported in the literature for peptides of other origins (Fitzgerald et al., 2012; He et al., 2013). Although the protein isolate was able to inhibit renin, its inhibitory capacity was more than 20 times lower than the hydrolysate, which again highlights the need to release encrypted peptides with greater potency than their original proteins. The hydrolysate exhibited a renin competitive inhibition behavior, with a Ki value of 0.25 mg/ml, which was lower than that obtained for the hemp seed hydrolysate (Girgih et al., 2011). Peptidic renin inhibitors from other origins have shown competitive, uncompetitive, non-competitive, and mixed inhibition mechanisms, implying the existence of binding sites other than the active site. These interactions modify the affinity of the active site for the substrate. Quiroga et al. (2017) also fractionated the isolate by molecular exclusion chromatography and then by RP-HPLC. The results achieved showed that the inhibitory activity was directly related to the hydrophobicity of the peptides. The most active fraction showed an inhibition of 67 ± 5%/mg of peptide. In this fraction, six peptides were identified: QAFEDGFEWVSKF, AFEDGFEWVSKF, SFNLPILR, FNLPILR, SFNLPIL, VNVDDPSKA containing almost the same amount of hydrophobic amino acids (about 43%), and hydrophilic (about 47%), the remaining 10% of total amino acids was P and G.

Using bioinformatics tools, Nardo et al. (2020) analyzed the interaction of the aforementioned peptides with renin. Initially, they used the CABS-dock server to perform a global docking assay on the enzyme surface, to obtain the renin-peptide complexes and to analyze the residues involved in the interaction. For each evaluated peptide, 10 models were generated which were analyzed. The authors found that the 6 peptides analyzed were capable of binding to different sites of the enzyme, including the active site, and that there were certain residues of the renin sequence that participated more frequently in the interaction, suggesting the presence of protein-protein recognition motifs located on the renin surface. Using the FlexPepDock server, they performed a local docking experiment and estimated the energies of the peptide-renin interaction only in the cases in which the peptide interacted with the active site of the enzyme (**Figure 2B**). The best interaction energies were achieved with the peptides AFEDGFEWVSKF and SFNLPILR. In order to validate the results achieved *in silico*, the authors carried out the chemical synthesis of these two peptides, as well as FNLPILR and VNVDDPSKA, and determined their renin inhibitory capacity *in vitro*. FNLPILR was the most potent inhibitor, with an IC_50_ value of 0.41 mM, and VNVDDPSKA was not able to inhibit the enzyme at any concentration in the range used by the authors. SFNLPILR and AFEDGFEWVSKF also showed a lower inhibitory activity, with IC_50_ values of 2.50 and 1.47 mM, respectively. These results did not totally agree with score values obtained with FlexPepDock, which predicted a higher affinity for SFNLPILR followed by AFEDGFEWVSKF and FNLPILR. Nardo et al. (2020) stated that these inconsistencies had already been reported by other authors, attributing the problem to the scoring function of the docking technique using peptides as ligands.

As the hydrolysate, FNLPILR acted as a competitive inhibitor. Moreover, the six peptides interacted with the renin active site in different ways. For example, SFNLPILR orients its N-terminal towards catalytic residues, while AFEDGFEWVSKF peptide interacts with the active site through the R residue located at its C-terminal. The latter peptide was found to establish two hydrogen bonds, and one π-interaction with renin residues. SFNLPILR interacts by SF motif, FNLPILR do it by LR motif.

Given that renin is the first enzyme in the RAAS system and that it is only capable of hydrolyzing angiotensinogen, its inhibition is key in the regulation of BP.

Unlike ACE, studies related to the inhibition renin are scarce, both in amaranth and in other food systems. Finally, the inhibition of renin could be another mechanism of action of amaranth peptides.

## Functional Foods

The existence of peptides encrypted in the food protein sequence bearing a variety of physiological activities opens up a very interesting possibility for the production of novel functional foods. On the other hand, this possibility presents new challenges related to the modifications that the elaboration processes can exert on such biological activities, as well as the presence of different components that can act as either activators or antagonists of the bioactive components.

Suárez and Añón (2019) have assessed two types of properties of amaranth proteins: their techno-functional properties and the presence of physiologically active sequences in their structure. The objective of their study was to elaborate 20:80 O:W emulsions formulated with equal amounts of protein isolates of *A. hypochondriacus* (API), and an alcalase hydrolysate (APH) (1–2% protein API50:APH50) as surfactants and carriers of bioactive peptides. The authors obtained highly flocculated emulsions that were stable upon creaming and coalescence for at least 8 days. According these authors, the peptides present in the hydrolysate could form part of the interfacial film and/or the floc network present in the continuous phase of the emulsion. The emulsions formulated with API were subjected to a simulated gastrointestinal digestion process. The results obtained showed an important stability of the emulsion during the gastric stage and the appearance of aggregation and coalescence phenomena in duodenal stage. In the digestive stage, a rapid release of fatty acids of the order of 29% was also detected, without any induction stage. This relatively low value was probably due to the accumulation of lipolysis products on the droplet surface, thus reducing enzymatic activity. The behavior of the API50:APH50 emulsion during the simulated gastrointestinal digestion process was similar to that described for API emulsions. Finally, these authors verified that the emulsions formulated before and after being subjected to the gastrointestinal digestion process had a dose-dependent ACE inhibitory effect (IC_50_ 0.29 ± 0.03 and 0.14 ± 0.02 mg/ml, respectively). The bioactive peptides responsible for this activity can be peptides present in the API and APH used in the formulation of the emulsion, which were not affected by the gastrointestinal enzymes and/or new peptides generated by the action of those enzymes from the same substrates. This type of bioactive emulsion displaying antihypertensive properties opens possibilities for the formulation of functional foods, such as dressings. Previously it was discussed that Suárez et al. (2020) have shown that these emulsions administered to Wistar SHR reduce their BP to normal values.

Valdez-Meza et al. (2019) have elaborated pastes, modifying the protein content by partial replacement of semolina by variable amounts of an amaranth protein concentrate and a constant amount of amaranth protein hydrolysate (IC_50_ 0.014 mg/ml). The control containing semolina had 11% protein, while the amaranth formulations, 15 and 20%. The addition of amaranth reduced cooking time and losses of pasta; increased its firmness and reduced the adhesiveness. Supplemented pastes, in turn, were less luminous and presented a more intense brown color than the control. On the other hand, amaranth supplementation negatively impacted the total acceptability of the products and the taste. The ingestion of the 8 g of cooked supplemented pasta decreased significantly systolic blood pressure of SHR Wistar rats after 3 h of ingestion, as compared to controls, and this effect was sustained for about 7–8 h. The authors attribute the antihypertensive effect of the product mainly to the addition of the hydrolysate to the matrix. Since supplemented pastes contain a significant amount of amaranth protein concentrate, both the proteins present in it and other components could contribute to the result achieved.

Sabbione et al. (2018)\ formulated amaranth flour cookies that are suitable for vegan and gluten-free diets and with adequate physicochemical and sensory characteristics. The cookies provide 114 Kcal per 30 g serving. The cookies and flour used were subjected to a simulated gastrointestinal digestion process, reaching 38 and 41% DH, respectively. The digests obtained displayed a dose-dependent ACE inhibitory activity *in vitro*. The IC_50_ values were 0.23 ± 0.03 and 0.08 ± 0.01 mg/ml for cookies and flour, respectively. Different IC_50_ values obtained for both samples could be attributed to the release of different peptides during simulated GID. Since the nature of proteins is the same in flour and cookies, the presence, in the latter, of a matrix and other components could modify the action of the different enzymes that act in the digestion process.

Ontiveros et al. (2020) formulated wheat flour cookies in which 10% of the wheat flour was replaced by a amaranth alcalase hydrolysate with proven hypertensive properties both *in vitro* and *in vivo* (Ramírez-Torres et al., 2017). Authors obtained cookies with adequate characteristics, which were administered to BALB/c mice and SHR rats in order to determine the bioavailability of the bioactive peptides and their action on the blood pressure. Their results showed that after 5 min of ingestion, the compounds reach the plasma and have an inhibitory activity on ACE that lasts 2 h. In addition, the intake of cookies causes a reduction of blood pressure after 3 h of ingestion, to reach a level similar to that achieved with captopril. This effect was maintained for up to 6 h after ingestion.

The results achieved so far show that the inhibitory potency of peptides studied is much lower than that of classical drugs such as captopril. Therefore, it is necessary much higher amounts of amaranth proteins than drug, to achieve an *in vivo* BP reduction equivalent to captopril. This is valid not only for amaranth peptides but also for peptides from animal and vegetable sources. In most of the *in vivo* tests performed the amount of protein given to the animals, either as flour, API, APH, emulsions, cookies or pastas was approximately 1.9–1.2 g/kg rat in a single intake, 38–25 times more than the unique peptide assayed (VIKP, 50 mg/kg rat) and 76–48 times more than the captopril dose administered (25 mg/kg rat). In order to have an approximation of the amount of amaranth protein that a human should consume we have used the human equivalent dose calculation (Center for Drug Evaluation and Research, 2005). According with these data 1.9–1.2 g/kg rat, would be equivalent to 0.30–0.19 g/kg humans. Considering the amount of protein present in the different protein sources this value is equivalent to, approximately 1.5–1.0 g of grains or flours, 0.47–0.3 g of API and 0.66–0.42 g of APH by kg human. These values must be transformed into quantity of food that will depend on their protein content.

It is evident that the presence of amaranth proteins added as flour, protein concentrates and isolates and hydrolyzed in different food matrices, allows obtaining different functional foods with antihypertensive activity. The combined use of bioactive and techno-functional properties of amaranth proteins open up very interesting perspectives for the development of novel products.

## Conclusions

The results depicted above showed that amaranth is a natural source of peptides and protein hydrolysates with antihypertensive activity, whose target can be located at the level of circulating and local RAAS. The main objective of the study of these peptides is to use them as an alternative to drugs and/or functional foods to reduce the risk of developing hypertension and/or complement conventional therapies.

In the case of amaranth peptides, to achieve this objective there are still studies related to the standardization of methods for obtaining the peptides, determination of the absorption mechanisms and identification of markers to track the peptide or its metabolites in animal models and/or humans. Moreover, further studies related to the signaling mechanisms that trigger these peptides, as well as clinical trials should be conducted to verify their health benefits. The usefulness of bioinformatics and computer tools should also be considered. More extensive molecular dynamics simulations are required to analyze, at the atomic level, the peptide-enzyme active site or peptide-receptor interactions. Likewise, the use of new algorithms based on technology such as machine learning can be extremely useful to identify new peptides from complex mixtures.

It is unlikely that antihypertensive peptides will replace conventional drugs in a short time, therefore the study and development of novel and attractive functional foods is of utmost importance. In the development of this type of food, the techno-functional properties of amaranth proteins should be taken into account, as well as the possible existence of peptides with more than one physiological activity or mechanism of action.

In addition, considering the more recent achievements related to the classical and local RAAS new studies should be focused in the natriuretic and vasodilator action that opposes the damaging effects of AngII. It is necessary to search for new peptides and/or to analyze the action of already identified peptides on the newer components of the RAAS system. The effect of peptides on the up-regulation of ACE2, blocking the AT1 receptor or the activity of peptidases that mediate the transformation of AngII into AngIII would be relevant to study (**Figure 1**).

Finally, a more holistic look should be taken on the blood pressure control system and consider the possible action of antihypertensive peptides on the levels of oxidative stress, inflammation, and prothrombotic effect.

In summary, to consider a food as functional and to be able to make a health claim on its label, many multidisciplinary studies are required. Particularly in the case of amaranth antihypertensive peptides, although clinical trials have yet to be conducted, large and promising advances have been made demonstrating that amaranth proteins are sources of antihypertensive peptides.

## Author Contributions

AN, SS, and AQ contributed their own results, built the figures and tables, organized the bibliography and contributed to the discussion and correction of the manuscript. MCA management starts with the results of this review and was in charge of organizing and writing it.

## Funding

Funding for this research was provided by Agencia Nacional de Promoción Cientíﬁca y Tecnológica (ANCyP, Argentina) PICT-20161537.

## Conflict of Interest

The authors declare that the research was conducted in the absence of any commercial or financial relationships that could be construed as a potential conflict of interest.
